# Gelation Based on Host–Guest Interactions Induced by Multi-Functionalized Nanosheets

**DOI:** 10.3390/gels7030106

**Published:** 2021-08-04

**Authors:** Hao Ding, Sana T. Khan, Jingjing Liu, Luyi Sun

**Affiliations:** 1 Polymer Program, Institute of Materials Science, University of Connecticut, Storrs, CT 06269, USA; hao.2.ding@uconn.edu (H.D.); jjl668@hotmail.com (J.L.); 2 Department of Chemical and Biomolecular Engineering, University of Connecticut, Storrs, CT 06269, USA; sana.t.khan@uconn.edu; 3 Department of Biomedical Engineering, University of Connecticut, Storrs, CT 06269, USA

**Keywords:** host–guest interaction, β-cyclodextrin, α-zirconium phosphate, gelation

## Abstract

Host–guest interaction, being reversible and stimuli-responsive, is ideal to be applied to the design of hydrogels. We created a gelation system based on the host–guest interactions between the adamantyl groups and β-cyclodextrin (β-CD) polymer. N,N,N-trimethyl-1-adamantylammonium hydroxide (TriMAA) cations were attached to the pre-exfoliated α-zirconium phosphate (α-ZrP) nanosheets by ionic bonding through a displacement reaction with the exfoliating agents. The exfoliated α-ZrP nanosheets with adamantyl groups directly or indirectly attached to the surface act as reversible high-functionality crosslinkers within the β-CD polymer. The gelation occurred at a host-to-guest ratio of 1:10 or 1:5 at room temperature within minutes. The agents used to exfoliate α-ZrP can tailor the surface of the resultant α-ZrP nanosheets and the ionic strength of the system, which directly affects the further gelation results. Plus, the exfoliating agent cations may generate a host-and-guest interaction with the β-CD polymer as well. This gelation process without covalent bonding formation should help fellow researchers to better understand the gelation system and host–guest interactions.

## 1. Introduction

Host–guest interaction usually refers to the formation of a complex between two species through special structure relationship and non-covalent bonding only [1]. A representative example is a crown-shaped hollow host molecule to interact with a guest molecule that could fit into the cavity of the host molecule [2]. The inclusion of the guest molecule into the host molecule is usually reversible and stimuli-responsive [3]. Therefore, host–guest interactions have drawn wide attention due to the broad applications in topological superstructures [4], sensing [5,6], drug delivery [3,7], catalysis [8], and many more fields [9,10,11]. One of the most used host molecules is cyclodextrin (CD) [12], and some novel supramolecular hydrogels based on the CD-based host–guest interactions have been developed [5]. The first method to achieve this is to use CD to interact with polymer chains directly to form poly(pseudo)rotaxanes [13]. Another method is to attach CD molecules and guest molecules onto polymer backbones, using the host–guest interactions as the weak crosslinking points to realize gelation [5]. A high-functionality crosslinker with host–guest interactions has the potential to form host–guest-based hydrogels [14].

α-Zirconium phosphate ((Zr(HPO_4_)_2_·H_2_O, α-ZrP) [15], being a layered compound with a high density of surface hydroxyl groups [16,17,18,19] suggests itself as a potential candidate for the template of high-functionality crosslinkers. Crystalline α-ZrP was first synthesized by Clearfield and Stynes via refluxing amorphous α-ZrP with phosphoric acid back to 1964 [15], and other synthesis methods to achieve an even higher crystallinity were then developed [20,21,22,23]. The layers of α-ZrP are constructed by zirconium atoms connected through the oxygen atoms of the phosphate groups. Three phosphate oxygens bond to Zr atoms, and the remaining phosphate oxygen bonded with a hydrogen, pointing into the interlayer space being a hydroxyl group [17,24,25,26]. This structure, with abundant hydroxyl groups, endows α-ZrP with a high ion exchange capacity of 6.64 meq/g [27,28], which also facilitates the intercalation of α-ZrP [29,30,31,32,33]. Bulk crystalline α-ZrP can be exfoliated by amines [34,35,36,37,38,39] into single-layer nanosheets with a thickness of below 1 nm but a diameter of several micrometers [16,21,40,41,42] to further expose the hydroxyl groups. The resultant exfoliated α-ZrP nanosheets, therefore, could be utilized as nanofillers for nanocomposites [43,44,45,46,47] and templates for creating abundant host–guest interactions for gels [48,49,50].

In this work, we designed an aqueous gelation system based on the abundant host–guest interactions distributed on exfoliated α-ZrP single-layer nanosheets and investigated the reversibility of the physical gelation under shaking.

## 2. Results and Discussion

**Figure 1:**
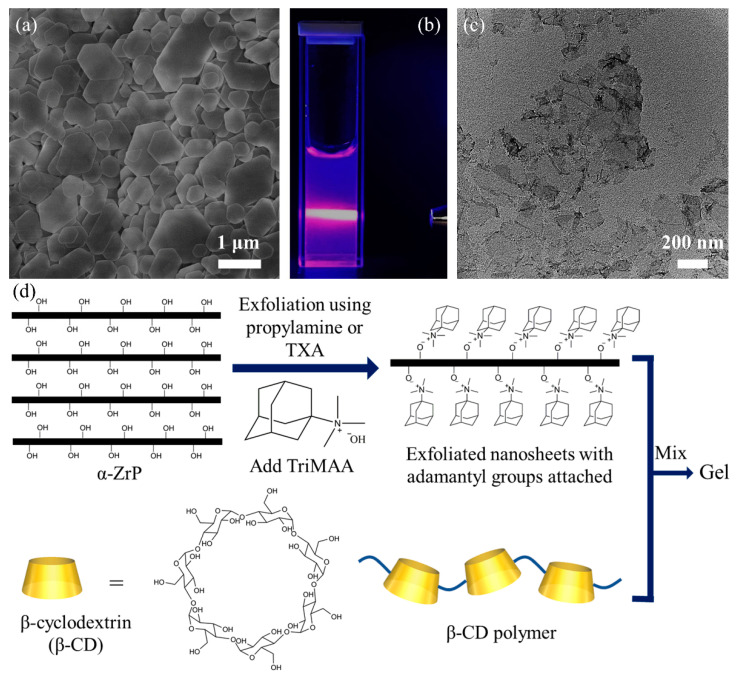
(**a**) Scanning electron microscopy (SEM) image of α-ZrP microcrystals; (**b**) digital photograph of the α-ZrP aqueous dispersion exfoliated by tetra(n-butyl)ammonium hydroxide (TBA) showing the Tyndall effect; (**c**) transmission electron microscopy (TEM) image of a TBA exfoliated α-ZrP nanosheet; (**d**) schematic of the design of a gelation system based on the host–guest interaction.

In this work, we proposed a design of a gelation system, making use of abundant host–guest interactions, as shown in Figure 1. α-ZrP microcrystals (Figure 1a) could be exfoliated by propylamine or TXA [36] in an aqueous system into single-layer nanosheets, forming a homogenous colloid dispersion (Figure 1b). The TEM image in Figure 1c shows the representative ZrP nanosheets exfoliated by TBA. The exfoliated nanosheets could react with TriMAA, a quaternary ammonium hydroxide possessing an adamantyl group. The resulting dispersion of nanosheets with adamantyl groups directly or indirectly attached to the surface led to gelation after being mixed with β-CD polymer (Figure 1d). This phenomenon is expected due to the high density of adamantyl groups on the surface of α-ZrP nanosheets, acting as guest molecules, and the abundance of hosting sites (β-CD) on the polymer chains in the dispersion. Even though no chemical crosslinkers were added into the aqueous dispersion, the numerous host–guest interactions between the nanosheets and the polymer chains, with the help of ionic bonding between the TriMAA and the α-ZrP nanosheets, will bind the polymer chains together and form a gel [51].

**Figure 2:**
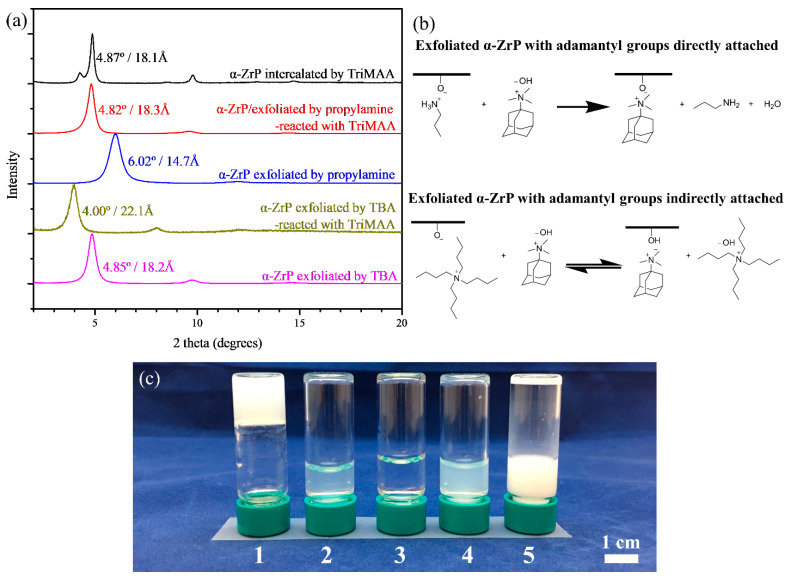
(**a**) XRD patterns of the exfoliated (n_ZrP_:n_exfoliating agent_ = 1:1 or n_ZrP_:n_exfoliating agent_:n_TriMAA_ = 1:1:1) and intercalated (n_ZrP_:n_TriMAA_ = 1:1) α-ZrP; (**b**) reactions of the exfoliated α-ZrP (using propylamine or TBA) with TriMAA; (**c**) Digital photograph of (1) 1-10-PA/β-CD polymer; (2) 1-10-PA(no TriMAA)/β-CD polymer; (3) 1-10-PA(no ZrP)/β-CD polymer; (4) 1-10-PA/β-CD monomer; (5) 1-10-PA(un-exfoliated ZrP)/β-CD polymer.

From the very beginning, we wanted to ensure the attachment of adamantyl groups onto the α-ZrP nanosheets. Because exfoliation of α-ZrP could be achieved with TXA [36], which are all quaternary ammonium hydroxides, it is natural to assume that TriMAA, with a similar structure and basicity, has the potential to exfoliate α-ZrP. However, after mixing TriMAA and α-ZrP, intercalation occurred, according to the peak from the XRD pattern (4.87°/18.1 Å) being significantly sharper than the restacked exfoliated α-ZrP nanosheets using propylamine (6.02°/14.7 Å) or TBA (4.85°/18.2 Å), as shown in Figure 2a. Therefore, the direct exfoliation of α-ZrP using TriMAA was not successful. Another approach to prepare exfoliated α-ZrP nanosheets with adamantyl groups attached is needed.

Considering that TriMAA is a stronger base than propylamine, one could imagine that TriMAA should replace the propylamine molecules that have already formed ionic bonding with the phosphate groups on the surface of the exfoliated α-ZrP nanosheets (Figure 2b). The interlayer distances of the TriMAA intercalated α-ZrP and the restacked propylamine exfoliated α-ZrP after reacting with TriMAA are 18.1 and 18.3 Å, respectively (Figure 2a). These two close interlayer distances support the replacement of propylamine molecules attached to the phosphate groups by TriMAA. On the other hand, when TBA was used as the exfoliating agent, this forward reaction, i.e., the displacement reaction between the TBA on the exfoliated α-ZrP and TriMAA, is not favored due to the similar basicity and bulkiness of these two ions (Figure 2b). The reaction tends to reach an equilibrium, with both ions staying on the surface of the nanosheets. As Figure 2a shows, the interlayer distance of the restacked sample of TBA-exfoliated α-ZrP after reacting with TriMAA is 22.1 Å, higher than that using either only TBA (18.2 Å) or only TriMAA (18.1 Å), which indicates the coexistence of the two types of cations on the surfaces, extra cations occupying more space and adding up the layer-layer repelling force. Note that in Figure 2a, minor peaks showing up at a large 2*θ* range are from the second or third order diffraction of the main peak, and therefore are not labeled.

Following the attachment of adamantyl groups onto the surface of α-ZrP nanosheets using various strategies, we then moved on to investigate their gelation behaviors. Besides sample 1-10-PA, several control samples missing specific interactions were designed and prepared to demonstrate the necessity of these interactions for gel formation. As Figure 2c shows, sample 1-10-PA formed gel with β-CD polymer as expected, while all the other samples absent specific interactions did not. Sample 1-10-PA in lack of TriMAA does not have the host–guest interactions between the nanosheets and the polymers. Sample 1-10-PA without α-ZrP and propylamine is the sample missing the nanosheets functioning as the high-functionality crosslinkers. 1-10-PA with hydroxypropyl-β-cyclodextrin (β-CD monomer) instead of β-CD polymer is the sample missing the polymer chains. For the last sample, 1-10-PA with unexfoliated α-ZrP, the exposure of surface functional groups on α-ZrP is limited, and hence very limited crosslinking effectiveness. Besides, unexfoliated α-ZrP could not disperse well in water.

**Figure 3:**
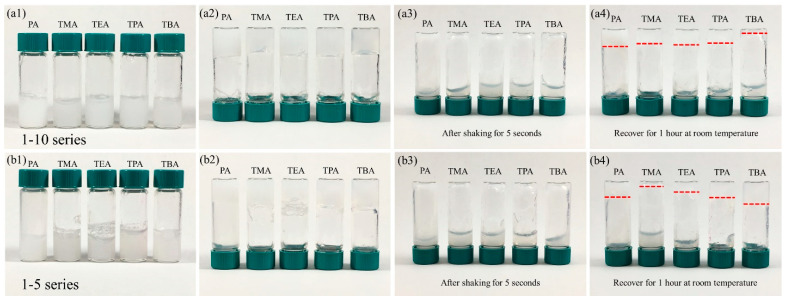
Digital photographs of the 1-10 series of (**a1**) as-prepared, (**a2**) as-prepared, with bottles inverted, (**a3**) after shaking for 5 s, with bottles inverted, (**a4**) after being left for 1 h at room temperature, with bottles inverted, and of the 1-5 series of (**b1**) as-prepared, (**b2**) as-prepared, with bottles inverted, (**b3**) after shaking for 5 s, with bottles inverted, (**b4**) after being left for 1 h at room temperature, with bottles inverted. The red dash lines show the remaining gels on the bottom.

To more systematically investigate this gelation system, we then explored the type of exfoliating agents used. Even though propylamine was selected to be the appropriate exfoliating agent according to the preliminary XRD results, it is meaningful to find other exfoliating agents that also cause gelation of the system. As shown in Figure 3a1,a2, no matter which exfoliating agent was selected, the 1-10 series samples all formed gels, which we believe results from the high ionic strength of the samples, especially for the TXA series. More interestingly, the gelation with TXA was much faster than the one with propylamine (several seconds vs. several minutes). This difference can be attributed to the addition of TXA, a stronger base compared to propylamine, which will cause an elevation in ionic strength of the dispersion; therefore, more electrostatic interactions accelerate the gelation.

The exfoliating agents chosen may affect the reversibility of the host–guest interaction-driven gelation and accordingly, as such, the reversibility of the gelation systems was also investigated. The samples were shaken for 5 s to return to a liquid state (Figure 3a3) and then left for recovery at room temperature for 1 h (Figure 3a4). Sample 1-10-PA demonstrated the reversibility to the most degree, while samples 1-10-TMA, 1-10-TEA, and 1-10-TPA all partially recovered but were slightly inferior to 1-10-PA. Nevertheless, 1-10-TBA barely recovered to be a gel. The affinity of TriMAA cations (guest species) to α-ZrP nanosheets was believed to be the critical factor for the reversibility of this physical gelation. TBA cations being the largest ones, compete with TriMAA cations the most under the confinement within the dispersion, severely interfering with: (1) the attachment of TriMAA cations to the nanosheets and (2) the host–guest interactions between TriMAA and β-CD polymer, which was supported by the ATR-FTIR spectra (Figure S1, in the Supplementary Material).

Another series of experiments (1-5) doubling the amount of hosting sites (β-CD polymer) was carried out to help better understand this gelation system. Like the 1-10 series, all the 1-5 samples formed gels completely at the beginning (Figure 3b1,b2). After shaking, all the samples returned to a liquid state (Figure 3b3). The intriguing part is the reversibility difference of the 1-5 samples. Sample 1-5-PA, as expected, went through gelation similar to 1-10-PA. For 1-5-TXA, the reversibility increases with the size of the TXA, with 1-5-TMA barely forming a hydrogel again and 1-5-TBA exhibiting the best reversibility (Figure 3b4). TXA anions affect the interface between the α-ZrP surface filled with guest groups and the host polymer. The TXA ions interact with β-CD, and the reactivity increases with the size of the TXA (TBA > TPA > TEA >> TMA) because the inner side of cyclodextrin is hydrophobic [52], and thus the longer the carbon chains, the more reactive TXA tends to be. Consequently, when the number of the hosts is doubled (the host-to-guest ratio changing from 1-10 to 1-5), the extra host groups enclose the larger TXA cations, decreasing the steric hindrance and improving the interface. Thus, the energy required for the recovery of hydrogel is lowered.

**Figure 4:**
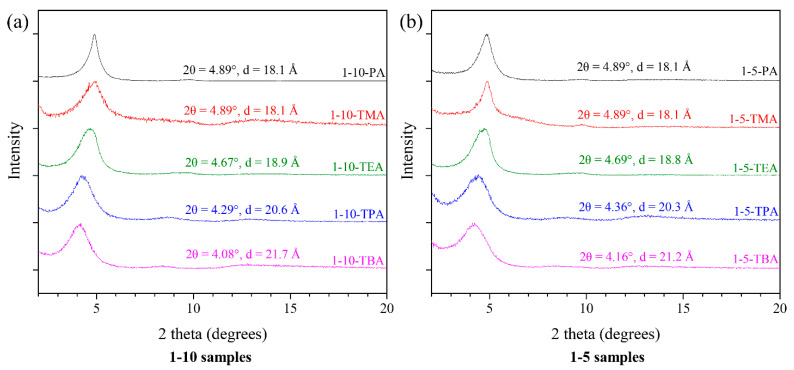
XRD patterns of the dropcast (**a**) 1-10 series and (**b**) 1-5 series.

The XRD patterns of the dropcast 1-10 and 1-5 series samples indirectly prove the host–guest interactions between the TXA cations and β-CD polymers (Figure 4). The interlayer distances of the dropcast 1-10-PA and 1-5-PA are both 18.1 Å, same as the interlayer distance of the TriMAA intercalated α-ZrP (Figure 2a), indicating that β-CD polymer chains cannot enter the restacked α-ZrP galleries. 1-10-TMA and 1-5-TMA have similar interlayer distances of 18.1 Å despite the stronger basicity of TMA compared to PA, which is possibly because of the smaller size of TMA. As a TMA cation is much smaller than a TriMAA cation, the interlayer distance change caused by TMA is not comparable to other TXA cations, which show a different trend. As shown in Figure 4a,b, doubling the amount of β-CD polymer (hosting sites) used, the interlayer distance of the samples all decreased slightly by 0.1, 0.3, and 0.5 Å for TEA, TPA, and TBA, respectively. The drop in interlayer distance indicates a partial removal of the cations from the surface of α-ZrP by the host–guest interaction with the β-CD polymer. This interlayer distance drop was more apparent when a larger exfoliating agent is used, implying that TXA cations could also interact with the β-CD polymer and be removed, as revealed above.

## 3. Conclusions

Adding TriMAA into the pre-exfoliated α-ZrP led to the α-ZrP nanosheets with adamantyl groups attached to the surface, which underwent gelation after mixing with β-CD polymer at a host-to-guest ratio of 1:10 or 1:5. The gelation relies on the host–guest interaction mainly, as well as the ionic bonding, the β-CD polymer, and the full exfoliation of α-ZrP. Exfoliating agents selected can tailor the surface of α-ZrP. According to the XRD patterns, when propylamine is used, adamantyl groups are directly attached to the surface of α-ZrP; when TBA is used, the high basicity and bulkiness cause the indirect attachment of adamantyl groups onto the nanosheets. Besides, the high basicity of the system brought up by TBA could raise the ionic strength of the dispersion and accelerate the gelation. More importantly, TXA cations may also interact with β-CD polymer through host–guest interactions, competing with TriMAA cations. This gelation system with tailorable reversibility controlled by the host-to-guest ratio and exfoliating agents selected is promising in sensing applications and could help fellow researchers better understand the host–guest interactions and hydrogel formation.

## 4. Materials and Methods

### 4.1. Materials

Zirconyl chloride octahydrate (ZrOCl_2_·8H_2_O, >98%, Acros Organics, Fair Lawn, NJ, USA), phosphoric acid (85%, Fisher, Pittsburgh, PA, USA), propylamine (PA; 99%, Acros Organics, Fair Lawn, NJ, USA), tetramethylammonium hydroxide (TMA; 25% in water, Acros Organics, Fair Lawn, NJ, USA), tetraethylammonium hydroxide (TEA; 25% in water, Acros Organics, Fair Lawn, NJ, USA), tetrapropylammonium hydroxide (TPA, 25% in water, Acros Organics, Fair Lawn, NJ, USA), tetra(n-butyl)ammonium hydroxide (TBA; 1.0 M in methanol, Alfa Aesar, Haverhill, MA, USA), hydroxypropyl-β-cyclodextrin (97%, Acros Organics, Fair Lawn, NJ, USA), and β-cyclodextrin (β-CD) polymer (average molecular weight 2000–300,000 Da, Sigma-Aldrich, St. Louis, MO, USA) were used as received without further purification. N,N,N-trimethyl-1-adamantylammonium hydroxide (TriMAA, 20% in water, grade ZeoGen™ SDA2820) was acquired from SACHEM (Austin, TX, USA).

### 4.2. Synthesis of α-ZrP

In a Teflon-lined pressure vessel, 4.0 g of ZrOCl_2_·8H_2_O and 40 mL of 6.0 M phosphoric acid were sealed and heated at 200 °C for 24 h [21]. After the reaction, the product was washed with water, ethanol, and water sequentially three times using a centrifuge. The sediment was dried at 70 °C for 24 h, and ground with a mortar and pestle into fine powders.

### 4.3. Preparation of α-ZrP Nanosheets with Adamantyl Groups Attached

#### 4.3.1. Direct Intercalation of TriMAA into α-ZrP

A sample of 0.0150 g of α-ZrP (0.050 mmol) was dispersed in 1.00 mL of water via ultrasonication, and 54.4 μL of TriMAA (0.050 mmol) was added to the dispersion, followed by further ultrasonication in an ice bath.

#### 4.3.2. Exfoliated ZrP with Adamantyl Groups Directly Attached

A sample of 0.0150 g of α-ZrP (0.050 mmol) dispersion in 1.00 mL of water was exfoliated by 0.50 mL of 0.10 M propylamine water solution (0.050 mmol) via ultrasonication in an ice bath. An amount of 54.4 μL of TriMAA (0.050 mmol) was added to the clear dispersion, followed by further ultrasonication.

#### 4.3.3. Exfoliated ZrP with Adamantyl Groups Indirectly Attached

A sample of 0.0150 g of α-ZrP (0.050 mmol) dispersion in 1.00 mL of water was exfoliated by 0.50 mL of 0.10 M TBA water solution (0.050 mmol) via ultrasonication in an ice bath. An amount of 54.4 μL of TriMAA (0.050 mmol) was added to the clear dispersion, followed by further ultrasonication.

### 4.4. Gelation Based on the Host–Guest Interaction

A sample of 0.0150 g of α-ZrP (0.050 mmol) dispersion in 1.00 mL of water was exfoliated by 0.50 mL of 0.10 M propylamine (or TMA, TEA, TPA, TBA) water solution (0.050 mmol) via ultrasonication. An amount of 54.4 μL of TriMAA (0.050 mmol) was added to the clear dispersion, and then 100 μL of β-CD polymer (0.050 or 0.100 M in terms of the concentration of β-CD monomers) water solution was added to the dispersion to form hydrogels with a host-to-guest molar ratio of 1:10 or 1:5, respectively, followed by a brief ultrasonication (5–10 s). The samples were left still at room temperature for gelation, which took seconds for the TXA (TMA, TEA, TPA, or TBA) samples and several minutes for the propylamine sample. The water content of all samples is ca. 98 wt.% The samples were labeled as 1-*x*-*y*, where *x* is the host-to-guest ratio (10 or 5), and *y* is the exfoliating agent used (e.g., 1-10-TBA means that the host-to-guest ratio is 1:10, and TBA was utilized to exfoliate α-ZrP).

### 4.5. Control Samples

#### 4.5.1. Gelation Tests Missing Guest Molecules

A sample of 0.0150 g of α-ZrP (0.050 mmol) dispersion in 1.00 mL of water was exfoliated by 0.50 mL of 0.10 M propylamine water solution (0.050 mmol) via ultrasonication. An amount of 100 μL of β-CD polymer (0.050 M in terms of the concentration of β-CD monomers) water solution was added to the dispersion with a host-to-guest molar ratio of 1:10, followed by a brief ultrasonication (5–10 s). The sample was left still at room temperature.

#### 4.5.2. Gelation Tests Missing α-ZrP Nanosheets

A sample of 0.50 mL of 0.10 M propylamine water solution (0.050 mmol) was diluted into 1.00 mL of water. An amount of 54.4 μL of TriMAA (0.05 mmol) was added to the solution, and then 100 μL of β-CD polymer (0.050 M in terms of the concentration of β-CD monomers) water solution was added to the clear solution with a host-to-guest molar ratio of 1:10, followed by a brief ultrasonication (5–10 s). The sample was left still at room temperature.

#### 4.5.3. Gelation Tests Using Host Molecule Monomers

A sample of 0.0150 g of α-ZrP (0.050 mmol) dispersion in 1.00 mL of water was exfoliated by 0.50 mL of 0.100 M propylamine water solution (0.050 mmol) via ultrasonication. An amount of 54.4 μL of TriMAA (0.050 mmol) was added to the clear dispersion, and then 100 μL of hydroxypropyl-β-cyclodextrin (0.050 M) water solution was added to the dispersion with a host-to-guest molar ratio of 1:10, followed by a brief ultrasonication (5–10 s). The sample was left still at room temperature.

#### 4.5.4. Gelation Tests with Unexfoliated α-ZrP

A sample of 0.0150 g of α-ZrP (0.050 mmol) was dispersed in 1.00 mL of water via ultrasonication. An amount of 54.4 μL of TriMAA (0.050 mmol) was added to the clear dispersion, and then 100 μL of β-CD polymer (0.050 M in terms of the concentration of β-CD monomers) water solution was added to the dispersion with a host-to-guest molar ratio of 1:10, followed by a brief ultrasonication (5–10 s). The sample was left still at room temperature.

#### 4.5.5. Reversibility of Gelation

The samples after gelation were shaken with hand for 5 s until the samples returned to the liquid state. The samples in the vials were then left at room temperature for 1 h and then turned over for gelation evaluation [53,54,55].

### 4.6. Structural Characterization

Scanning electron microscopy (SEM) images were taken on an FEI Nova NanoSEM 450 microscope. The samples were sputter-coated with a gold/palladium (80/20) layer (ca. 10 nm) before imaging. Transmission electron microscopy (TEM) images were recorded on a Talos 200 S/TEM, operated at 100 kV. The TBA-exfoliated ZrP dispersion containing nanosheets was dropcast on a 400-mesh copper grid with a carbon supporting film before imaging. X-ray diffraction (XRD) patterns were recorded on a Bruker D2 phaser (30 kV and 10 mA), using a nickel monochromator with Cu-Kα radiation. The samples in aqueous dispersions were dropcast on silicon wafers for XRD characterization. Attenuated total reflection Fourier transform infrared (ATR-FTIR) spectra were acquired on a Nicolet Magna 560 spectrophotometer on dropcast samples.

## Data Availability

Not applicable.

## Supplementary Materials

The following are available online at https://www.mdpi.com/article/10.3390/gels7030106/s1, Figure S1. ATR-FTIR spectra of β-CD polymer, TriMAA, and 1-10-PA.
