# Supplementary material for: Gelation Based on Host–Guest Interactions Induced by Multi-Functionalized Nanosheets
Source: Gels. 2021 Aug 4;7(3):106. doi: 10.3390/gels7030106 (PMC8396050; doi:10.3390/gels7030106)
Supplement: Supplementary file 1 [file gels-07-00106-s001.zip › gels-1306341-supplementary.pdf]

Supplementary Materials

Gelation Based on Host–guest Interactions Induced by Multi-functionalized Nanosheets

Hao Ding,[1,2] Sana T. Khan,[2] Jingjing Liu,[1] Luyi Sun[1,2,3,*]

[1]Polymer Program, Institute of Materials Science, University of Connecticut, Storrs, Connecticut 06269, United States; hao.2.ding@uconn.edu (H.D.); jjl668@hotmail.com (J.L.)

[2]Department of Chemical and Biomolecular Engineering, University of Connecticut, Storrs, Connecticut 06269, United States; sana.t.khan@uconn.edu (S.K.)

[3]Department of Biomedical Engineering, University of Connecticut, Storrs, CT 06269, United States

*Correspondence: luyi.sun@uconn.edu

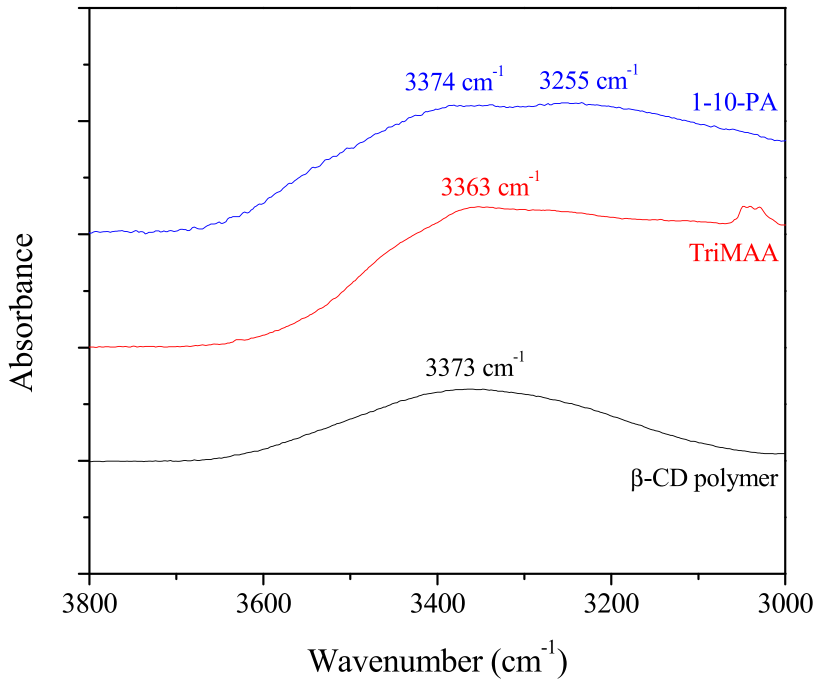

Figure S1. ATR-FTIR spectra of β-CD polymer, TriMAA, and 1-10-PA.

The peak position of -OH vibration can be used as an indication of the host–guest interaction through the hydrogen bond.[1] The wavenumber of -OH vibration in β-CD polymer and TriMAA are 3373 and 3363 cm^{-1}, respectively. However, within the 1-10-PA gel, besides the peak at 3374 cm^{-1}, another broad peak showed up at 3255 cm^{-1}, which indicates the formation of aggregation compound.[2]

1. Zhou, C.; Cheng, X.; Zhao, Q.; Yan, Y.; Wang, J.; Huang, J. Self-assembly of nonionic surfactant tween 20@ 2β-CD inclusion complexes in dilute solution. *Langmuir* **2013**, *29*, 13175–13182.
2. Zhong, X.; Hu, C.; Yan, X.; Liu, X.; Zhu, D. Aggregate morphology transition of an adamantane-containing surfactant via the host–guest interaction with β-cyclodextrin. *Journal of Molecular Liquids* **2018**, *272*, 209–217.
